# “Good luck, social distance”: rapid decarceration and community care for serious mental illness and substance use disorder during the COVID-19 pandemic

**DOI:** 10.1186/s40352-023-00238-5

**Published:** 2023-09-18

**Authors:** Jennifer E. James, Emily F. Dauria, Riya Desai, Adelaide Bell, Jacob M. Izenberg

**Affiliations:** 1 Institute for Health and Aging, University of California, San Francisco, San Francisco, CA USA; 2 School of Public Health, Department of Behavioral and Community Health Sciences, University of Pittsburgh, Pittsburgh, PA USA; 3 School of Medicine, University of California, San Francisco, San Francisco, CA USA; 4 Washington and Lee University, Lexington, VA USA; 5 Department of Psychiatry and Behavioral Sciences, University of California, San Francisco, San Francisco, CA USA

## Abstract

The COVID-19 pandemic inspired calls for rapid decarceration of prisons and jails to slow the spread of disease in a high-risk congregate setting. Due to the rarity of intentionally-decarcerative policies, little is known about the effects of rapid decarceration on individuals with serious mental illness (SMI) substance use disorder (SUD), a population who receive many services via the criminal legal system (CLS). We conducted interviews with 13 key informants involved in CLS in San Francisco, CA to better understand the implication of the decarcerative policies put into practice in early 2020. Participants described a tension between the desire to have fewer people incarcerated and the challenges of accessing services and support – especially during the lockdown period of the pandemic – outside of the CLS given the number of services that are only accessible to those who have been arrested, incarcerated, or sentenced. These findings emphasize the need for investing in community social services rather than further expanding the CLS to achieve the goal of supporting individuals with SMI and SUD shrinking the US system of mass incarceration.

## Introduction

The COVID-19 pandemic inspired calls for decarceration, the release of people from detention, as a public health measure (Macmadu et al. 2020). Occurring in the context of a broader movement to unwind the policies promoting mass incarceration in the US, pandemic-driven decarceration efforts led to considerable jail and prison census reductions over a short period, primarily in 2020 (The-Impact-of-COVID-19-on-Crime-Arrests-and-Jail-Populations-JFA-Institute.pdf. n.d.). Prior to this, little was known about the effects of rapid decarceration on vulnerable populations; its unique challenges notwithstanding, this time period offers an important case study in both the potential for and policy considerations surrounding decarceration.

Jails are locally run facilities that typically house people who are awaiting a disposition of criminal charges or serving short sentences; most of the roughly 550,000 individuals incarcerated in US jails at any given time will return to the community within a fairly short timeframe (Initiative and Wagner 2022); (James and Glaze 2006). Jails have a high prevalence of serious mental illness (SMI) and substance use disorders (SUDs), with 34% of recently-arrested detainees meeting criteria for a depressive, psychotic, or bipolar illness or PTSD in one survey, (Steadman et al. 2009) and 68% of those in jail meeting criteria for a SUD in the year before arrest according to another estimate (Karberg and James 2002). This situation is the product of social and political trends spanning nearly a century, including underfunded and inadequate psychiatric services, welfare state retrenchment, and laws criminalizing homelessness and addiction (Dvoskin et al. 2020; Lurigio 2011).

The disproportionate representation of individuals living with SMI and/or SUDs in jail has contributed to the rise of interventions that attempt to leverage community behavioral health as a means of reducing incarceration. (Ollove 2015). Examples include mental health courts (MHCs), in which those facing criminal charges can elect to undergo supervised mental health treatment in exchange for reduced or dismissed charges, (Schneider 2008) as well as the mental health treatment often provided through probation or other supervised release.

### Decarceration in San Francisco, CA

As with many places in the US, (The-Impact-of-COVID-19-on-Crime-Arrests-and-Jail-Populations-JFA-Institute.pdf. n.d.) San Francisco (SF), California saw an unprecedented decrease in its jail census early in the COVID-19 pandemic. While pandemic-related public health prerogatives sparked this rapid change, San Francisco was already poised to advance these efforts. In late 2019, the city elected Chesa Boudin, a reform-minded public defender, as District Attorney (DA). Table 1 outlines local policies and executive orders that shaped San Francisco’s approach to decarceration, beginning after DA Boudin took office in 2020.

**Table 1:** Local policies and executive orders shaping San Francisco, CA’s approach to decarceration, 2020 (Doyle 2020; Initiative n.d.)

Policy	Enacted date	Description
**No Cash Bail (**Boudin 2020).	**January 20, 2020**	San Francisco, CA. New policy passed by District Attorney (DA) Chesa Boudin, forbids prosecutors from requesting money bail under any circumstances. It also allows prosecutors to request pretrial detainment only for people who have certain violent charges and who are believed to pose a high risk of violence or flight
**Shelter-In-Place** (City and County of San Francisco 2020)	**March 17, 2020**	San Francisco, CA. This order passed by Mayor London Breed, requires that San Franciscans stay home except for essential needs. The initial order was placed into effect until April 7, 2020
	**March 24, 2020**	San Francisco,CA. The Director of Jail Health Services (Dr. Lisa Pratt), called for a reduction of the jail population to mitigate the risk of a COVID-19 outbreak by permitting increased flexibility for isolation, quarantine, and social distancing within the facility
**Zero Bail**	**April 6, 2020**	California. A state-wide emergency bail schedule reduced the bail to $0 for most misdemeanor and some low-level felony cases to reduce jail population with the hope of slowing the spread of COVID-19. While effective in reducing jail populations, the policy was rescinded on June 20, 2020. (Balassone 2020)
**Closing Prisons and Youth Lockups to New Admissions (**California and of. 2020)	**April 13, 2020**	California. By executive order (N-36-20), Governor Gavin Newsom closed California’s 35 prisons and four juvenile detention facilities to new admissions or transfer for 30-days, to slow the spread of COVID-19

When SF instituted COVID-19 shelter-in-place order in March 2020, 1,100 people were incarcerated in the county jail. Following calls from local leadership to reduce the population in jails, the DA’s Office prioritized the release of individuals identified as being the most vulnerable, while seeking to institute other ways of reducing jail incarceration. Efforts included avoiding low-level charges, reserving pre-trial detention for those with high risk of flight or great bodily harm to others, identifying candidates for temporary housing and reentry support, and expediting linkage to community-based mental health and substance use treatment. At the same time, the State of California adopted a 30-day freeze on transferring people newly sentenced from county jails to state prisons while the state’s Judicial Council mandated zero-dollar cash bail for many low-level offenses. Through this combination of local and state policy, San Francisco was able to reduce the jail census to a low of 699 on April 24^th^; a 40% reduction relative to Jan 2020 (Macmadu et al. 2020).

Although the pandemic reshaped the conditions of community life faced by those leaving carceral settings, COVID-19 nevertheless created a sort of decarceration stress-test, including for those with SMI, SUDs, and other behavioral health needs. In this context, we undertook a qualitative study of key informants involved in early pandemic decarceration efforts in San Francisco. Using this major US city as a case study, we sought to better understand stakeholders’ perceptions of the implementation of decarceration strategies for jail-incarcerated individuals with serious behavioral health needs.

## Methods

### Participants and recruitment strategy

To be eligible for participation, individuals had to: 1) be ≥ 18 years, 2) be a stakeholder in decarceration efforts or work for the jail, the judicial system, city behavioral health services, or reentry services; and 3) speak English. Our team used purposive sampling methods to generate an initial list of potential informants from our professional networks and invite them to participate via email. Participants were asked to refer additional potential participants. Sampling was driven by a desire to interview participants who worked across community mental health, the jail system, and the court system. In total, 24 participants were asked to participate. 11 participants did not respond after two emails were sent. Recruitment ended after participants were recruited from across all service sectors involved in decarceration in San Francisco.

### Procedures

Data were collected between July 2020 and March 2021. Semi-structured qualitative interviews were conducted by phone or Zoom and lasted approximately 60 min. Semi-structured interviews were used to allow participants to describe their experiences in detail, in a private setting where they could share their perspectives on the policies and practices of their and other departments and agencies. Interviews were completed by two members of the research team (JEJ and JI) and verbal consent was obtained prior to the start of the interview. Interviewers followed a semi-structured interview guide exploring informants’ views of how rapid decarceration impacted individuals with SMI and/or SUD. Participants were asked about their role before and during the pandemic, how they or their office were involved in setting decarceration policy, and to reflect on what decarceration policies looked like in practice. After each interview, a short interviewer-administered questionnaire was completed to assess each participants’ age, professional experience, gender, and race and ethnicity (Table 2). Participants were not paid for their study participation.

**Table 2:** Sociodemographic Characteristics of Stakeholders (*N* = 13)

	**Total**
	***N* = 13**
	**Median (range) or N (%)**
Median age (years)	49.4 (31-60)
Gender	
Man	7 (53.8%)
Woman	6 (46.2%)
Race/Ethnicity	
White	10 (76.9%)
Latinx	1 (7.7%)
Asian	1 (7.7%)
Multiracial	1 (7.7%)
Years of employment^a^	13.0 (3 – 30)

^***a***^Years of employment was self-reported by ***n*** = 11

### Analysis

All interviews were audio-recorded, transcribed verbatim and de-identified. Informed by Inductive Thematic Analysis, (Braun and Clarke 2006) the initial codebook was developed after a round of open coding the first three interview transcripts, including in-vivo from the participants’ responses (e.g., “criminalization versus medicalization”). Interviews were coded by four trained members of the study team. After independently coding each transcript, coders met to review codes and resolve discrepancies in order to improve reliability and refine coding patterns. Consensus was reached in code list and coding approach across all interviews. After intitial coding was complete, coded segments were reviewed, and JEJ created memos to highlight connections between codes and subcodes relevant to the study’s primary research questions. We compiled coded quotations and developed concepts and relationships pertinent to core themes. The final set of codes and memos were compared and combined into overarching themes and subthemes. Themes were discussed, refined, and named for the final analysis. We used Atlas.ti qualitative data analysis software (Berlin, Germany) to facilitate qualitative analysis.

### Ethics

Study protocols were approved by the University of California, San Francisco Institutional Review Board.

## Findings

We conducted 13 stakeholder interviews with individuals working in the jail health and behavioral health services, the public defender’s and DA’s offices, probation department, and sheriff’s office, as well judges and community health workers. Most participants self-identified as being white (76.9%) and male (53.8%) and reported working in their field for an average of 13 years (*range:* 3 to 30 years) (Table 2). Next, we present findings from our qualitative interviews, first highlighting the pathway through which the jail serves as a link to behavioral service access. We then describe the implementation of decarceration in San Francisco. Lastly, we outline one case study offered by participants highlighting the ideal pathway to service referral and linkage post-decarceration.

### “Not available to people otherwise”: Behavioral Health Service Linkage from Carceral Settings Before and After the COVID Pandemic

In San Francisco, the county jail system is closely linked to other systems of care and social support. Probation is well funded and represents a main pathway to services for many with SMI or SUD. During probation, individuals have access to resources that are “not available to people otherwise.” As one participant working within the court stated, “if somebody’s on probation or not can be the difference between whether they're eligible for housing or not, or certain therapy or not.” Prior to the pandemic, reentry service providers were able to enter the jail and work directly with clients set to be released to help them establish linkages to care prior to release.

System stakeholders described how the COVID-19 pandemic led to several shifts in where, how, and for how long individuals had contact with jails and subsequently behavioral health services. San Francisco’s COVID policies (Table 1) meant that fewer people were able to go into the jails to facilitate release planning and linkage to health and social services. Consequently, at a time when more people were being released from jail there were fewer resources and support to coordinate a safe release plan.

The crisis of the pandemic increased demand for SUD and SMI treatment services in the city at a time when those on the front lines were scrambling to deliver services in the context of emergent public health requirements (e.g., social distancing). Participants noted that shelters, navigation centers, and other social service providers were not accepting new clients in the first few months of the pandemic, leading to gaps in service provision; fewer patients who were receiving treatment for SUD and SMI were being discharged from inpatient facilities because there wasn’t a safe, socially distanced plan in place. Participants described not having time or resources to make support plans for their clients. As one person involved in reentry planning described, “I ended up having to get boxes of tents from the homeless coalition. And I've never in my life had to give somebody leaving jail a tent and say, ‘good luck, social distance.’” Additionally, participants described how connections to SMI and SUD services were limited in the first months of the pandemic. As one participant involved in the diversion courts described, “it’s very hard to do any kind of outpatient treatment right now.”

Participants perceived that those who *were* able to access SUD and SMI-related programming, often did well, despite the pandemic or even in some cases because of it. One respondent involved in the treatment courts hypothesized that, “maybe they were more successful because…there was less staff hassling them. They didn't have to come to court as much. They didn't have as many people telling them what to do and where they had to be.” This respondent noted how individualized this was and that if a client was motivated, intervention was possible in this environment. Others agreed, with the caveat this was only true for those who could access programming; the number of individuals eligible for either inpatient or outpatient treatment decreased as fewer people were entering the courts and the jails. As one respondent working within the jail noted,

“As far as drug treatment, unfortunately with the deincarceration, these people are not receiving the services that they used to receive in the system. Basically, they are coming in for two or three days and detoxing and then getting diverted out to community services, which we all know just aren't there. There's not enough beds, there's not enough people out there addressing it on the street in face-to-face contact.”

### Decarceration in practice

Respondents described decarceration as influencing the entire carceral system, beginning with policing. As an attorney recalled, “There was also a good amount of pressure on the police department like, ‘Stop arresting and booking people for this minor, minor stuff.’ And so for a little bit, the police were actually using more discretion.” The zero-bail policy, which was mandatory statewide in Spring 2020, was described as a “blessing” by some, but others expressed more complicated feelings about it. One attorney noted,

“It’s so needed right now…. But, you know, it saddened me because those individuals really needed to have some sort of intervention. But the zero-bail order didn't allow for that. Because if they were arrested for another offense that qualified for zero-bail, the sheriff had to release.”

A law enforcement official echoed this concern, describing the implications of fewer people being arrested or many staying in jail for shorter periods of time:

“[Law enforcement is] not arresting as many people for the [penal code] 11364 possession of crack pipes, [penal code] 11350 possession of narcotics. They're not even coming to jail… Anybody who has a problem or an addiction they are not getting into a program quicker…they're falling through the cracks and going right back to the street.”

He is describing a counternarrative to the oft-cited concept of medicalization as an alternative to criminalization. In the absence of criminalizing SMI and SUD, this line of reasoning holds, these individuals may be *less* likely to receive care and treatment. This respondent is asserting that jail and the criminal legal system (CLS) are the primary pathway to medicalized services for this population. Without carceral contact and subsequent referral to diversion programs, many of these individuals end up not being able to access any services at all.

This was especially concerning for clients with comorbid conditions. One community health worker described one of her clients who has AIDS in addition to SMI and SUD, noting,

“every time I see him, I’m just so worried he’s going to die. I know that he can’t get 5150’d [involuntary psychiatric hold] because the bar for that is too high… I kind of want him to get arrested so that he can be in custody, stabilize, get clean and sober, and be on antiretrovirals [treatment for HIV/AIDS].”

This was echoed by other participants who noted that often the most straightforward way for a client to access services is through jail or probation.

Participants’ concern about decarcerative policies did not come from a consciously punitive mindset; rather there was concern about what they viewed as an inability to effectively provide services to an individual who may be in need. As an attorney described, “Let's say, John Doe who keeps getting out on zero bail keeps coming back again. There could be services rendered through the sheriff's reentry programs. They could intervene in the jails to offer this individual a place to stay or treatment.” In this example, the absence of jail time and access to sentencing diversion programs would lead this John Doe to go without needed care and services; our participants understood the criminal-legal system as a defacto safety net for behavioral care service delivery.

### Jail and probation as key features of the safety net

For many key informants, the sudden decarceration caused a confrontation between the ideal of having fewer people in jails and the realities of the lack of alternative services available to those who may be a danger to themselves or others. Consequently, many participants felt conflicted about the role that incarceration does or should play in providing services to those with SMI and SUD. One community health worker, articulated this saying,

“I love the theory of people not being incarcerated. But there are some of my clients who needed to be incarcerated because they were a very, very clear threat to themselves and others. It’s kind of heartbreaking … [when] cops see them and they’re like, ‘No we’re not going to do anything. You just very, very seriously hurt yourself and someone else. But both of you are homeless. No one’s gonna file a charge and I don’t want to do the paperwork. If we take you to custody like you're just going to get released anyway in a few hours.’ And one of those clients died of an overdose.”

This respondent is articulating that, due to jail serving as an involuntary safety net and the only avenue available to access services, when a client dies it feels like a death that could have been prevented by interventions that can only be accessed via CLS involvement. After their engagement with the decarceration process during the COVID-19 pandemic, many identified gaps in the safety net that were being patched by the jail and noted the essential function jail had come to serve in the lives of so many. At the same time, these participants acknowledged that the provision of these services or interventions comes at a great cost for the individual, with one community-based social worker wondering, “This person might die in the next few weeks… So, is it okay to advocate for their agency to be taken away because you’re afraid that they are going to die?”.

Another participant pushed back against the reliance on jail for behavioral interventions noting,

“A lot of people, especially people who are on the clinical side, want to keep people in custody because they are worried about them and they think they'll be safer there. I think you really have to resist that urge. So, I remind myself all the time: Jail is not detox. Jail is not treatment, right? Jail is incarceration. That's all.”

Yet, this tension remains. In San Francisco, as in most places, jail is utilized for more than incarceration. What is clear is that neither incarcerating someone nor releasing someone will necessarily solve problems related to SMI and SUD. This is only further compounded by the absence of residential treatment beds and other services for those in crisis, both before and during the pandemic. As one respondent within the court systems noted,

“there are people approved to go to residential treatment. The jail health people have said they’re ready to go, that they’re a good candidate, at the appropriate baseline level, and they wait months in custody for that bed to become available. And while they’re waiting, bad shit happens. On the other hand, if [they get] out and…wait for a bed out of custody, bad shit happens.”

Again, respondents described feeling forced to choose between incarceration and homelessness for their vulnerable clients. 

Participants described an internal conflict between their beliefs that jails are not safe environments and the sudden realization of the reliance of their clients and the county on the CLS to intervene and offer services. As one case manager described, “I think that the problem with [the district attorney] just like letting everyone out is that – I love that idea – but we don't have enough community resources in place to catch all those people and support them. And it is pretty traumatic to just be let out and have to figure out life. Especially if you've been institutionalized for a long time.”

The unique landscape of San Francisco led many participants to wonder if the implications of decarceration might be different in other counties, noting that, in San Francisco, “the bar to be incarcerated was already pretty high.” One participant expressed that there was already a narrower set of crimes for which people were being incarcerated, leading them to state: “I don't think that decarceration has been helpful, sadly. I don't want to say that, but I don't think it's helpful.” One community health worker noted that, “the clients that have more serious mental health issues are the ones that were impacted by deincarceration or not arresting people. It's those clients really that are, I think, harmed the most.” There remained a concern that this population had been abandoned. One respondent involved in community supervision, who themselves is in substance use recovery, noted, “as addicts we are promised three things: jails, institutions, or death. San Francisco has taken the first two off the table, so the only thing left is death, right?” While neither jails or institutions were working well as a safety net, for the most vulnerable they were all that was available. She went on to say, “People who’ve never been justice involved, they think, ‘oh, the minute somebody gets out of incarceration, everything’s better because the problem is them being locked up.” She is asserting that incarceration, while problematic, isn’t the core problem. Rather, it is a symptom of some of the most challenging societal ills, and those problems are not solved by releasing someone from jail without a change to the structure of community-based behavioral health services.

### The transformative potential of rapid decarceration

While many respondents described challenging experiences of decarceration there was one case that multiple participants highlighted as an example of a clear success. This case involved “Mr. F,” an older adult who had spent several years incarcerated in a San Francisco jail. Mr. F had been living with mental illness prior to incarceration, and during his detention was diagnosed with dementia and cancer. The reasons for his incarceration meant that most residential facilities would not accept him as a client, leaving him waiting in jail. Recognizing his profound vulnerability to COVID as the pandemic’s risks to incarcerated people became clear, multiple stakeholders worked together to coordinate this individual’s release and linkage to community-based behavioral health services by identifying funds to pay for a private board and care for one year. One participant described Mr. F’s post-release situation by stating that despite being quite ill, Mr. F was “out. And he's happy. And he has a space. And he has dignity, and clothes, and, you know, the stuff he didn't have here. So, I see that as a huge success.” This stakeholder identified the resources applied to Mr. F’s case as somewhat novel for the various parties involved.

This scenario highlights how decarcerative policies in San Francisco in 2020 could lead to the successful release and service linkage for an individual with SMI and other complex needs. Central to this success was that key stakeholders collaborated to pool ideas and resources and find an appropriate non-carceral setting for an individual who was vulnerable to COVID-19. Mr. F. was able to be successfully released due to a team committing new resources for community care and working creatively and collaboratively in ways made possible by a shared sense of urgency.

## Discussion

The US system of mass incarceration has become a chronic, complex public health issue that touches virtually every US community (Wildeman and Wang 2017). The COVID-19 pandemic bought to light an acute crisis in jails and prisons (Barnert et al. 2021) and galvanized efforts to decarcerate. Decarceration is a critical public health goal (Reinhart and Chen 2020) and the pandemic created an opening, through newfound political will and a prioritization of public health imperatives, to reduce jail and prison populations. In San Francisco’s County Jail system, as in many other places, such a reduction did indeed occur, yet our findings indicate that decarceration is not simple in practice.

Respondents in our study called attention to the ways in which, access to social services, treatment and care have in many cases become inextricably linked to the carceral system; tying services explicitly to involvement in the CLS has created a paradox in which those who are most marginalized are dependent on the CLS for social interventions. The key informants we interviewed, many of whom support decarceration in principle and share the vision of a world in which many fewer people are incarcerated, were conflicted when confronted with a very sudden push towards decarceration without the social safety net provided by the CLS, particularly at a moment when the pandemic severely undermined the availability and accessibility of an already inadequate service environment. Our findings demonstrate the many challenges that occur when the bars of a jail cell serve as the safety net; it is not a soft place to land and widening the gaps between the bars, a critical public health and human rights intervention, leaves many vulnerable when there are no other safety net systems in place to catch them.

Respondents in our study described the tension between mass incarceration as a public health crisis and jail as an opportunity to link individuals to treatment and other services, through both voluntary referrals and treatment that is court-mandated as a condition of bail, probation, or diversion from criminal legal sanctions. In some cases, courts may condition pre-trial release on a referral to or placement in a mental health or substance use treatment program or, with MHCs, participation in community-based care may be a pathway to reduced or dismissed criminal charges. While a review of the effectiveness of such interventions is beyond this scope of this paper, it should be noted that the evidence for MHCs for both reducing future CLS exposure and improving long term mental health is limited (Honegger 2015).

Many respondents in our study called attention to the lifesaving potential of such programs; in many cases, incarceration (or the threat thereof), creates an initial opportunity for respite and stabilization from active substance use or psychiatric instability or a fast-track to services. This situation points to the fundamental failure of the community mental health system, which has come to *rely* on CLS involvement in the case of vulnerable individuals. Unfortunately, those who agree to treatment in order to evade incarceration are often shuffled back into a dysfunctional mental health system marked by a chronically disjointed and inadequate supply of care and resources (Insel 2022). One particularly problematic outcome of this is the use of jail as a waiting room for appropriate community treatment; precisely the sort of situation these approaches are meant to alleviate.

### Limitations

There are several limitations to the present study that warrant discussion. First, we collected data from system stakeholders who work within the CLS and whose jobs were directly impacted by decarceration policies and practices in San Francisco, CA. Stakeholders were recruited using a combination of purposive sampling methods and therefore may not reflect the experiences of all system stakeholders. We did not collect data from individuals living with SMI or SUD describing their experiences during reentry, including their experiences being referred, linked, and engaging in community-based behavioral services. This decision was made for several reasons including that this was an unfunded study and we deemed it unethical to recruit this population without offering compensation. Future research should explore where and how decarceration practices and policies shape behavioral healthcare access and engagement among this population from the perspectives of those who have experienced incarceration. Similarly, the majority of our participants were white. While our sample does reflect the staff of the agencies from which we sampled and while many of our participants reflected quite thoughtfully on the role of race and racism in their perspectives on the criminal legal system, the whiteness of our sample in contrast to the racial demographics of those leaving the jail system is a limitation of both our study and the system overall. This study focused on policy and practice in San Francisco and our findings may not be transferable to other counties or outside of the US. Lastly, there has been a recent political backlash to decarceration and rebound in the jail census. Future research should explore whether and how decarceration policies and programs that were instituted and then rescinded shaped behavioral health access.

## Conclusion

Our research demonstrates the downstream challenges and consequences of enmeshing health services within the CLS. Through interviews with stakeholders working on the front lines during pandemic-related decarceration, we learned that community health workers have come to rely on incarceration as an opportunity for their clients to reset, seek sobriety, and reestablish connections to initiate case management and services. This reliance makes it difficult to provide services in the absence of criminal-legal involvement and makes it harder to work towards a system where we are incarcerating fewer people. MHCs and other diversion programs have merit in seeking to avoid the worse outcome of imprisonment. However, when the carceral system is leveraged as a mental health safety net, it is easy for this approach to be viewed as the answer to a crisis of community mental health care, rather than patch for a desperate situation. Rather, it is clear from our research that more investment, energy, and imagination need to be put into building systems of care that do not rely on the coercive power of the carceral system. We must decouple mental and behavioral health interventions from the CLS invest in a community, rather than carceral, safety net.

## Data Availability

The qualitative data generated and analyzed during the current study are not publicly available due their potential identifiability. Participants did not consent to having their data used for additional research projects. Portions of the data may be available from the corresponding author on reasonable request.
